# Surgical outcomes of two kinds of demineralized bone matrix putties/local autograft composites in instrumented posterolateral lumbar fusion

**DOI:** 10.1186/s12891-021-04073-3

**Published:** 2021-02-17

**Authors:** Dong-Gune Chang, Jong-Beom Park, Yangjun Han

**Affiliations:** 1 Department of Orthopaedic Surgery, College of Medicine, Inje University Sanggye Paik Hospital, Inje University, Seoul, South Korea; 2 Department of Orthopaedic Surgery, College of Medicine, The Catholic University of Korea, Seoul, South Korea; 3 Department of Orthopaedic Surgery, Uijeongbu St. Mary’s Hospital, College of Medicine, The Catholic University of Korea, Korea, 271 Cheonbo-ro, Uijeongbu-si, Gyeonggi-do 11765 South Korea

**Keywords:** Demineralized bone matrix, Local autograft, Composite, Instrumented posterolateral fusion, Bone graft substitute

## Abstract

**Background:**

This study aimed to assess the surgical outcomes of two kinds of demineralized bone matrix (DBM) putties/local autograft composites in instrumented posterolateral lumbar fusion (PLF).

**Methods:**

Twenty-seven fusion segments of 19 patients, who underwent decompression and instrumented PLF for lumbar spinal stenosis or degenerative spondylolisthesis less than grade 1, were included in this study. The PLF mass consisted of different two kinds of DBMs (Grafton® and DBX®) and local autograft. Next, 7.5 cc of Grafton® DBM/local autograft composite was implanted on the left side, and the same amount of DBX® DBM/local autograft composite was implanted on the right side in the same patient. The PLF masses of 54 total sides (27 Grafton® sides and 27 DBX® sides) were assessed for fusion based on both flexion/extension lateral radiographs and computed tomography images at 12 and 24 months postoperatively. Clinical symptoms were also evaluated.

**Results:**

At 12 months postoperatively, the fusion rates for the Grafton® and DBX® sides were 59.5 and 51.9%, respectively; the difference was not statistically significant (*P* = 0.425). At 24 months postoperatively, the fusion rates for the Grafton® and DBX® sides increased to 70.4 and 66.7%, respectively, but the difference was still not statistically significant (*P* = 0.574). Diabetes mellitus, smoking, and obesity (body mass index ≥25) negatively affected the fusion rate of both the Grafton® and DBX® sides. Visual analog scores for lower back pain and leg pain and Oswestry Disability Index were significantly improved after surgery (both, *P* < 0.01). No deep or superficial infections occurred postoperatively. No patients underwent revision surgery due to nonunion during follow-up.

**Conclusions:**

Our results suggest that two kinds of DBMs/local autograft composites might be considered as useful bone graft substitute in instrumented posterolateral fusion for lumbar spinal stenosis or degenerative spondylolisthesis less than grade 1.

## Background

Lumbar spinal fusion procedures are usually performed for lumbar degenerative diseases when preoperative instability is present or when postoperative instability is expected due to the extensive decompression required during surgery [1–8]. The ultimate aim of lumbar spinal fusion procedures is to achieve solid fusion at the index level. Ideal graft materials or graft substitutes should have three basic biologic attributes: osteogenicity, osteoconductivity, and osteoinductivity [9–18]. An autologous iliac crest bone graft (ICBG) is the gold standard for lumbar spinal fusion procedures because the substantial amount of cancellous bone that can be obtained from the inner table of the pelvis provides all the desired graft properties [18–30]. However, the morbidity rates associated with autologous ICBG use are high, with some studies reporting up to a 40% rate of persistent donor site pain, paresthesia, hematoma, and infection [11–14]. Potential alternatives to autologous ICBG, including local autograft, calcium-phosphate salts, demineralized bone matrix (DBM), the bone morphogenetic proteins (BMP) family, autogenous growth factors, bone marrow aspirate, and collagen base matrices, are gaining popularity and are being increasingly used in lumbar spinal fusion procedures [1–7]. Recently, the osteoinductive potential of allogeneic DBM has been studied as an alternative bone graft materials. There are several commercially available DBMs according to the carriers, especially hyaluronic acid (DBX®) carriers and glycerol carriers (Grafton®) and the form of the carrier choice of DBM may cause a difference of surgical outcomes.

It is vital to achieve a solid union following lumbar spinal fusion procedures because pseudarthrosis often causes disappointing clinical outcomes and often necessitate further revision surgery [8–10]. However, much controversy exists between fusion rates and clinical outcomes because a successful bony union does not always produce improved clinical outcomes [31, 32]. To the best of our knowledge, no prior studies have undertaken side-by-side comparisons of the fusion efficacy of two types of DBMs/local autograft composites for instrumented posterolateral lumbar fusion (PLF) in the same patients.

## Methods

### Study design

The inclusion criteria for this study were (1) spinal stenosis or degenerative spondylolisthesis less than grade 1 without instability; (2) a minimum follow-up period of 24 months after surgery; (3) follow-up radiology with plain radiographs including flexion/extension lateral radiographs and computed tomography (CT) both 12 and 24 months postoperatively; and (4) clinical assessment 12 and 24 months postoperatively. Exclusion criteria were (1) a history of previous spine surgery, (2) the presence of degenerative spondylolisthesis greater than grade 1, and (3) PLF with interbody fusion. Of the 22 patients, finally a total of 19 patients met the inclusion criteria and included in the study, whereas 3 patients were excluded due to lack of follow-up data.

In the current study, we used and assessed the fusion efficacy of Grafton® (Medtronic Sofamor Danek, Memphis, TN, USA) and DBX® (Musculoskeletal Transplant Foundation, Edison, NJ, USA) DBMs/local autograft composites in instrumented PLF procedures. From November 2016 to October 2017, 22 patients underwent decompression and instrumented PLF using Grafton® or DBX® DBMs/local autograft composite for one- to three-level surgeries from L2–3 to L4–5. All patients complained of lower back pain and leg pain with claudication that was unresponsive to conservative treatment for at least 3 months. The diagnosis was based on clinical symptoms, plain radiographs (including flexion/extension lateral radiographs), CT, and magnetic resonance imaging (MRI). Nineteen patients met all the inclusion criteria and were included in the study. However, three of these patients were subsequently excluded because they did not undergo follow-up CT performed within 24 months postoperatively. All data were collected prospectively but were retrospectively analyzed to characterize the fusion efficacy of two DBMs/local autograft composites for instrumented PLF. This research was approved by the Institutional Review Board of the Uijeongbu St. Mary’s Hospital. All participants agreed with the data and publication of the manuscript and all participants provided written informed consent. All declarations were performed in accordance with the relevant guidelines and regulations.

### Demographic data

Demographic data for the 19 patients are summarized in Table 1. The mean age at the time of surgery was 65.7 years (range: 43–82). Eight patients were male, and 11 were female. Thirteen patients underwent one-level surgery; four patients had two-level surgery, and two patients underwent three-level surgery. The majority of one-level fusions were performed at the L4–5 level (11 patients). The other two one-level fusions took place at the L2–3 and L3–4 levels. All four two-level fusions were performed at the L3–5 levels, and both three-level fusions were performed at the L2–5 levels, respectively. Eight patients (42%) had diabetes mellitus (DM), and four (21%) were current smokers. The mean body mass index (BMI) was 24.9 (range: 20.1–30.2); nine patients (47%) had a BMI < 25, and 10 patients (53%) had a BMI ≥ 25, which was considered obese including overweight. The mean follow-up period length after surgery was 33.8 months (range: 24–48 months).

**Table 1:** Demographic data for Grafton® DBM/local autograft and DBX® DBM/local autograft composites for instrumented posterolateral fusion

Mean age (years)	65.7 (range: 43–82)
Sex (male/female)	8/11
Mean follow-up (months)	33.8 (range: 24–48)
Surgery (fused segment)	19
One-level	13 (58%): 11 L4–5, 1 L3–4, 1 L2–3
Two-level	4 (21%): 4 L3–4-5
Three-level	2 (11%): 2 L2–3–4-5
Diabetes mellitus	8 (42%)
Smoking	4 (21%)
Mean BMI	24.9 (range: 20.1–30.2)
BMI < 25	9 (47%)
BMI ≥25	10 (53%)
Pedicle screw system	19
Optima™	12 (63%)
CD Horizon Legacy™	4 (21%)
Iliad™	3 (16%)

*DBM* Demineralized bone matrix, *BMI* Body mass index

Two DBMs/local autograft composites.

All surgeries were performed by a single senior spine surgeon. Local autobone was harvested from the spinous process, lamina, and facet joints during decompression and was cleaned of any remnant soft tissue and minced into pieces. The local autobone was divided evenly per side and per fusion segment. The PLF mass consisted of DBM and local autograft at a 1:2 ratio. The average amount of local autograft used per side was 5 cc (range: 4–6 ccs). For left-side fused segments, 2.5 cc of Grafton® DBM was combined with local autograft to form a composite graft. A 7.5-cc Grafton® DBM/local autograft composite was placed in the posterolateral gutters to bridge the intertransverse process space. For right-side fused segment(s), 2.5 cc of DBX® DBM was prepared in the same manner as the Grafton® DBM/local autograft composite, and 7.5 cc of DBX® DBM/local autograft composite were implanted during the same procedure in the same patient. No additional graft material, graft extenders, or enhancers were used. This unique study design with two DBMs implanted simultaneously in the same patient enabled each patient to serve as his or her own control.

### Fusion assessment

**Fig. 1:**
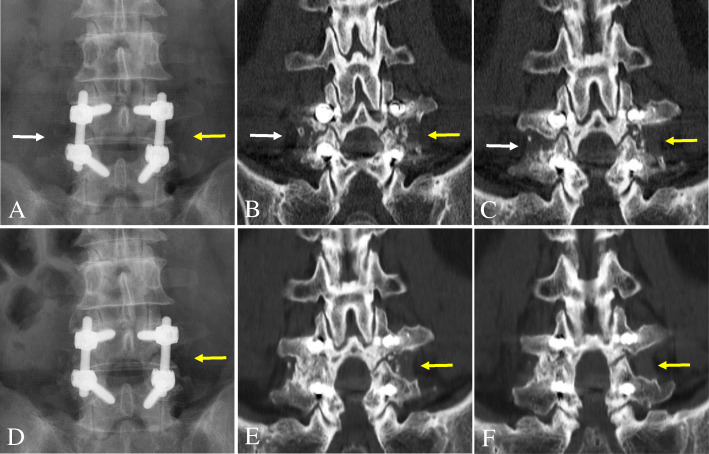
A 60-year-old male patient underwent decompression and instrumented posterolateral fusion using Grafton® (left side) and DBX® (right side) DBM/local autograft composites at L4–5. At 12 months postoperative, both sides showed nonunion (white and yellow arrows) on anteroposterior radiograph (**a**) and coronal two-dimensional computed tomography images (**b** and **c**). At 24 months postoperatively, the DBX® side was defined as a fusion, but the Grafton® side was labeled as a nonunion (yellow arrows) on anteroposterior radiograph (**d**) and coronal computed tomography images (**e** and **f**)

**Fig. 2:**
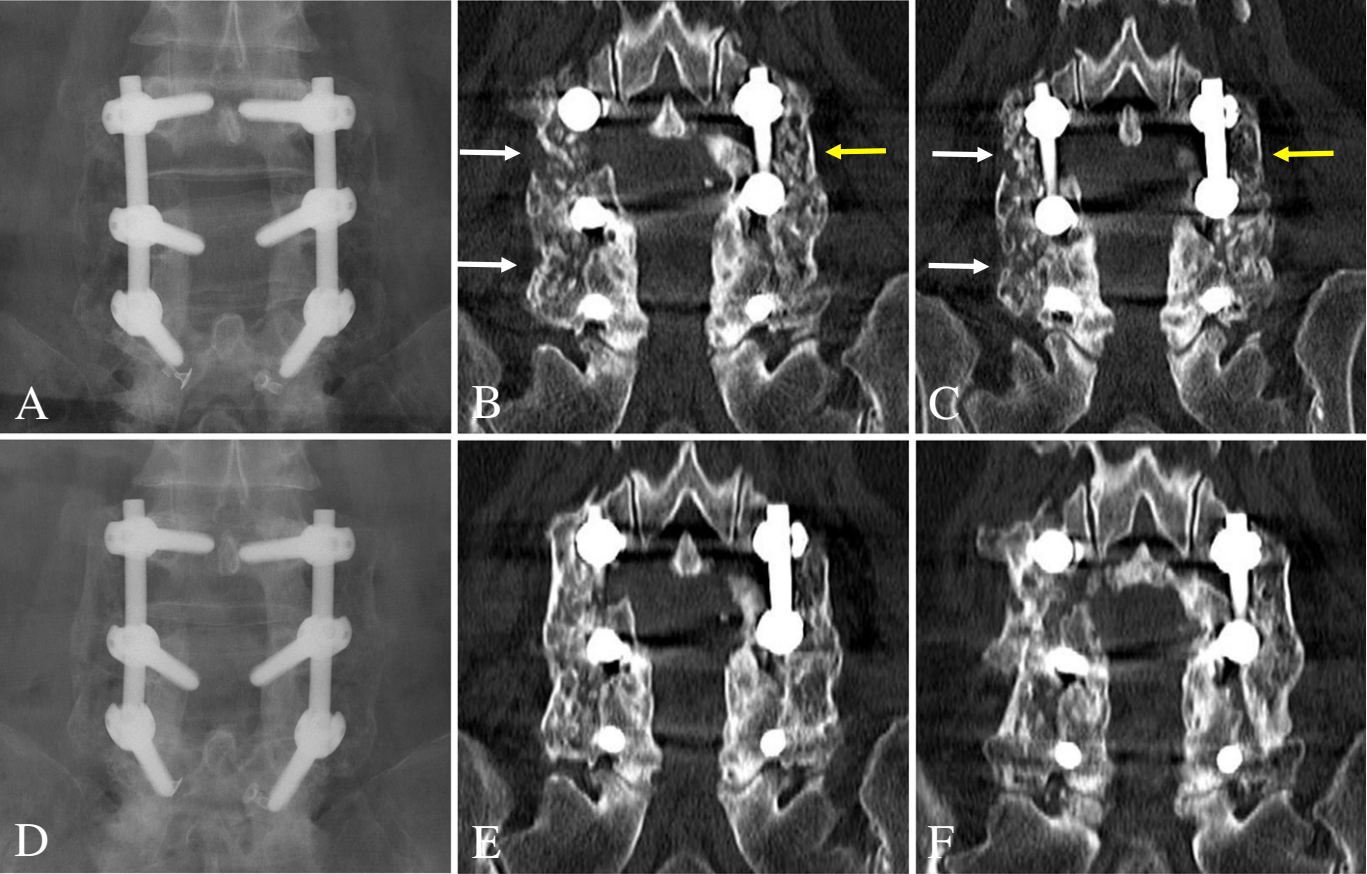
A 69-year-old female patient underwent decompression and instrumented posterolateral fusion using Grafton® (left side) and DBX® (right side) DBMs/local autograft composites at L3–4-5. At 12 months postoperative, L3–5 on the DBX® side (white arrows) and L3–4 on the Grafton® side (yellow arrow) were defined as nonunions on anteroposterior radiograph (**a**) and coronal two-dimensional computed tomography images (**b** and **c**). At 24 months postoperatively, L3–5 on both sides was defined as fusion on both anteroposterior radiograph (**d**) and coronal computed tomography images (**e** and **f**)

**Fig. 3:**
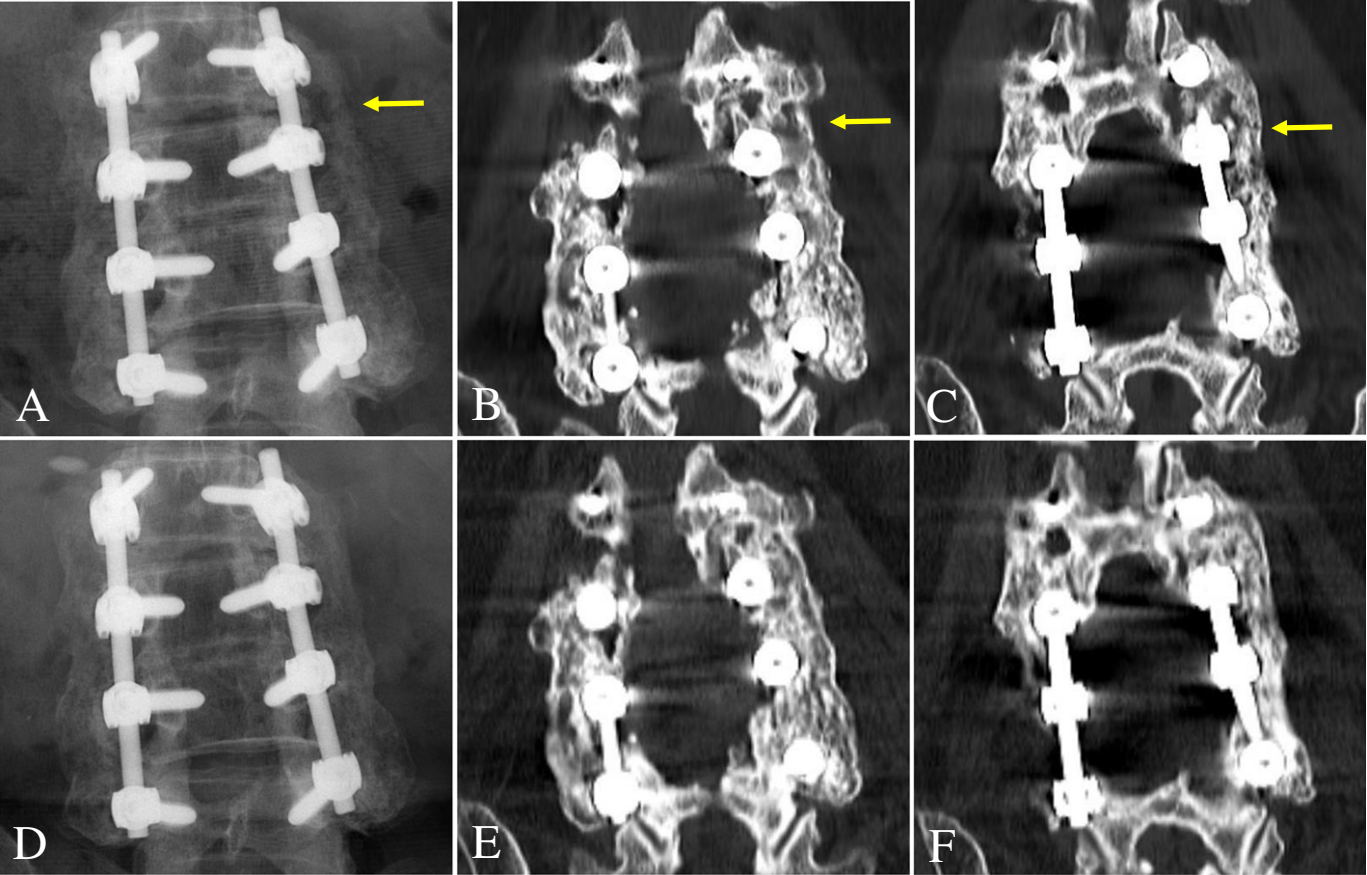
A 68-year-old female patient underwent decompression and instrumented posterolateral fusion using Grafton® (left side) and DBX® (right side) DBMs/local autograft composites at L2–5. At 12 months postoperatively, L2–5 on the DBX® side was defined as a fusion, but L2–3 on the Grafton® side (yellow arrow) was defined as a nonunion on anteroposterior radiograph (**a**) and coronal two-dimensional computed tomography images (**b** and **c**). At 24 months postoperatively, L2–5 on both sides were defined as fusions on both anteroposterior radiograph (**d**) and coronal computed tomography images (**e** and **f**)

The patients were routinely followed up with plain radiographs that included flexion/extension lateral radiographs at 6 weeks and then at 3, 6, 12, and 24 months postoperatively. Additionally, a 1-mm slice coronal CT was assessed to more accurately evaluate the fusion status after PLF at 12 and 24 months postoperatively. To determine the fusion status, two independent and blinded spine surgeons evaluated the postoperative anteroposterior and flexion/extension lateral radiographs and CTs. If the surgeons’ assessments did not agree, a third spine surgeon evaluated the films, and his assessment was used as the final result. Three criteria were used to determine fusion success at the surgical level: (1) < 3° of angulation on flexion/extension lateral radiographs, (2) < 2 mm of translation on flexion/extension lateral radiographs, and (3) bridging bone connecting the transverse process on consecutive 1-mm coronal images from CT. The fusion mass lateral to the instrumentation on each side of the fusion segment was independently judged as being fused or not (Fig. 1). Therefore, 54 total sides (27 Grafton® sides and 27 DBX® sides) of 27 fusion segments in 19 patients were evaluated (Fig. 2). Fusion was only deemed successful if all three criteria were met, which is a very strict standard compared with those used in previous studies (Fig. 3) [8, 11, 14]. Finally, we examined the effects of DM, smoking, and obesity (BMI ≥ 25) on the fusion status for the two DBM/local autobone composite grafts 24 months postoperatively.

### Clinical evaluation

A 10-point visual analog scale (VAS) was administered to evaluate lower back pain and leg pain preoperatively, at each follow-up time point, and again at 24 months postoperatively. The Oswestry Disability Index (ODI) was used to evaluate any pain and disability caused by the spinal condition preoperatively, at each follow-up time point, and at 24 months postoperatively.

### Statistical analysis

A Chi-square test and paired t-test were used for the statistical analyses. *P*-values < 0.05 were considered statistically significant.

## Results

The fusion rates for the two DBMs/local autograft composites for instrumented PLF are summarized in Table 2. At 12 months postoperative, the fusion rates of the Grafton® and DBX® sides were 59.5% (16/27 fusion segments) and 51.9% (14/27), respectively. Although the fusion rate of the Grafton® side was slightly higher than that of the DBX® side, this difference was not statistically significant (*P* = 0.425). At 24 months postoperative, the fusion rates of the Grafton® and DBX® sides had increased to 70.4% (19/27 fusion segments) and 66.7% (18/27), respectively. Although the fusion rate of the Grafton® side was slightly higher than that of the DBX® side, this difference was not statistically significant (*P* = 0.574).

**Table 2:** Comparative analysis of fusion rate of Grafton® and DBX® DBMs/local autograft composites at 12 and 24 months after instrumented posterolateral fusion

	Fusion Rate (%)		*P*-value
	Grafton® side (*n* = 27)	DBX® side (*n* = 27)	
Postoperative 12 months	59.5% (16/27)	51.9% (14/27)	*P* = 0.425
Postoperative 24 months	70.4% (19/27)	66.7% (18/27)	*P* = 0.574

*DBM* Demineralized bone matrix

DM, smoking, and obesity negatively affected the segment fusion status of both DBMs/local autograft composites 24 months after instrumented PLF (Table 3). Diabetic (55.6% vs. 75.0%, *P* < 0.01), smoking (58.3% vs. 71.4%, *P* < 0.05), and obese (≥ BMI = 25; 46.7% vs. 87.5%, *P* < 0.01) patients had lower fusion rates compared to non-exposed patients.

**Table 3:** Negative effect of diabetes mellitus, smoking, and obesity on fusion status of Grafton® and DBX® DBMs/local autograft composites in instrumented posterolateral fusion

	Fusion Status (*N* = 54; 27 Grafton® side + 27 DBX® side)		*P*-value
	Fused	Not Fused	
Diabetes mellitus			*P* < 0.01
No (*n* = 36)	75.0% (27/36)	25.0% (9/36)	
Yes (*n* = 18)	55.6% (10/18)	44.4% (8/18)	
Smoking			*P* < 0.05
No (*n* = 42)	71.4% (30/42)	28.6% (12/42)	
Yes (*n* = 12)	58.3% (7/12)	41.7% (5/12)	
Obesity^a^			*P* < 0.01
BMI (< 25) (*n* = 24)	87.5% (21/24)	12.5% (3/24)	
BMI (≥25) (*n* = 30)	46.7% (16/30)	53.3% (14/30)	

^a^Obesity including both overweight and obesity; *DBM* Demineralized bone matrix, *BMI* Body mass index

Clinical symptoms significantly improved after surgery (Table 4). The preoperative VAS scores for lower back pain and leg pain were 4.9 ± 1.7 and 7.2 ± 2.1, respectively; at 24 months after surgery, the VAS values decreased significantly to 2.1 ± 0.7 and 1.9 ± 0.8, respectively (*P* < 0.01 for both). Preoperative ODI was 42 ± 14.0, which decreased significantly at 24 months postoperative to 21 ± 6.0 (*P* < 0.01). No deep or superficial infections occurred postoperatively. No patients underwent revision surgery due to nonunion during follow-up.

**Table 4:** Clinical outcomes of Grafton® DBM/local autograft and DBX® DBM/local autograft composites in instrumented posterolateral fusion

	Preoperative	Postoperative 24 month	*P*-value
VAS			
Lower back pain	4.9 ± 1.7	2.1 ± 0.7	*P* < 0.01
Leg pain	7.2 ± 2.1	1.9 ± 0.8	*P* < 0.01
ODI	42 ± 14	21 ± 6	*P* < 0.01

*DBM* Demineralized bone matrix, *VAS* Visual analog scale, *ODI* Oswestry Disability Index

## Discussion

DBM is an acid extraction product of cadaver bone that was first developed by Marshall Urist in 1965 [33]. While most bone graft substitutes and synthetics are osteoconductive rather than osteoinductive, DBM has osteoinductive properties with the potential to aid in spinal fusion [21]. There are several commercially available DBM substances that are currently used in spinal surgery with different concentrations of osteoinductive proteins [21, 22]. The carrier choice is one important factor because it can affect the fusion efficacy and/or rate. As opposed to the neutral pH of hyaluronic acid (DBX®) carriers, negative effects have been observed with the use of glycerol carriers (Grafton®), which generate a highly acidic environment for host tissues, especially when used in large quantities at the fusion site [24, 34]. These previous studies have shown that commercially available DBM products exhibit significant variability in fusion performance, which is secondary to their differences in carrier medium and processing [24, 34].

DBM has been studied as a bone graft extender, enhancer, and substitute in animal models and has demonstrated significant intra-and inter-product performance variability [1–15]. However, there is little formal clinical evidence of its efficacy in instrumented PLF surgery [35–39]. Furthermore, much controversy exists regarding fusion rates and clinical outcomes because a successful bony union does not always result in improved clinical outcomes [31, 32]. Therefore, we tried to suggest a guideline for obtaining improved fusion rates and good clinical outcomes in the treatment of spinal stenosis or degenerative spondylolisthesis less than grade 1 without instability using DBM/local autograft composite without ICBG. To the best of our knowledge, no studies have undertaken a side-by-side comparison of the fusion efficacy of two kinds of DBMs/local autograft composites for instrumented PLF in the same patients. Therefore, we performed the current study to assess the fusion efficacy of two kinds of DBMs (Grafton® and DBX®)/local autograft composites in instrumented PLF using a side-by-side comparison.

Regarding the fusion rate, our study found no significant differences between the two DBMs/local autograft groups at 12 and 24 months postoperatively, even though the fusion rate of the Grafton® side was slightly higher than that of the DBX® side. In a Level I prospective multicenter randomized clinical trial, Kang et al. [14]. evaluated the efficacy of a DBM preparation (Grafton®) compared with an iliac crest autograft for one-level posterior lumbar fusion. The arthrodesis rate was evaluated using plain radiographs and CT scans and was 86% in the DBM group and 92% for the autograft group (*P* = 1.0), which indicated that the fusion rates and clinical outcomes associated with DBM were comparable to those of an iliac crest autograft. In their Level II study, Cammisa et al. [8] investigated the role of DBM as a fusion extender in conjunction with an autograft in 120 patients who underwent instrumented PLF. In each patient, an iliac crest autograft was implanted on one side of the spine, and a DBM (Grafton®)/autograft composite was implanted on the contralateral side. The authors concluded that DBM was an effective graft extender because it decreased the amount of autograft required and potentially reduced the risk for and severity of donor-site morbidity, however special attention would be needed to the fusion rate when performing the instrumented PLF with DBMs/local autograft composites only. Vaccaro et al. [38] conducted a Level II prospective study that evaluated DBM (Grafton®) use in instrumented PLF in 19 patients along with supplemental bone grafting with DBM putty enriched with aspirated bone marrow, 27 patients who had DBM putty combined with an iliac crest autograft, and 27 control-group patients who received only an autograft. After 24 months, 63% of the levels in the DBM/bone marrow group, 70% of the levels in the DBM/iliac crest group, and 67% of the levels in the autologous ICBG group demonstrated radiological fusion on anteroposterior, lateral, and flexion/extension radiographs (*P* = 0.875).

In this study, we used DBM combined with local autograft harvested from the spinous process, lamina, and facet joints during decompression. Many studies have reported that DBM combined with autologous laminectomy bone and osteoconductive materials is as effective as an autologous iliac bone graft at achieving long multi-segment posterolateral fusion success [11, 39, 40]. Through the combination of DBM and local autograft, custom bone graft composites can provide all three components necessary for bone formation: osteogenesis, osteoinduction, and osteoconduction. This combination can be used as an effective bone graft substitute for multi-segment PLF and may decrease morbidities associated with autogenous ICBG [11].

Many factors have an impact on fusion success, including surgical technique, primary or revision surgery, instrumentation, grafting materials, and patient comorbidities [41, 42]. Several studies have highlighted the deleterious effects of advanced age, osteoporosis due to slowed bone metabolism, and a decreased differentiating capacity of osteoprogenitor cells [1–3]. Our study showed that DM, smoking, and obesity negatively affected the fusion status of both DBMs/local autograft composites at 24 months after instrumented PLF. Therefore, it is important to consider these comorbidities when selecting the DBM/local autograft composite type for instrumented PLF to ensure the best fusion rate and improve surgical and clinical outcomes.

Some authors have reported a discrepancy between bone union status and clinical outcomes, and a successful bony union does not always lead to satisfactory clinical outcomes [31, 32]. In addition, there is controversy over the fact that successful fusion guarantees good clinical results. In some cases, successful fusion can often cause pain, while other patients with non-unions do not report pain. Fischgrund et al. [31] found that successful arthrodesis occurred in 83% of the instrumented PLF procedures. However, successful fusion was not predictive of a successful patient outcome. Herkowitz et al. [32] reported that pseudarthrosis was seen in about 36% of patients, but the clinical results were excellent; their study concluded that the development of a fibrous union appeared to provide sufficient structural support to prevent progressive spondylolisthesis. Similarly, our study showed comparable clinical outcomes showing successful response to conservative treatment despite the relatively lower fusion rates of about 70% after instrumented PLF. This finding may be due to the inclusion of study patients with spinal stenosis or degenerative spondylolisthesis less than grade 1 and fibrous union using a minimally invasive surgical technique with preservation of the facet joint capsule, which resulted in relatively good clinical outcomes even if a fibrous union occurred instead of a solid union. We concluded that the fusion efficacies and clinical outcomes of Grafton® and DBX® DBM/local autograft composite were improved after instrumented PLF for lumbar spinal stenosis or degenerative spondylolisthesis less than grade 1, which is a good indication for the use of DBM/local autograft composite without ICBG. However, careful caution should be pay attention to its use in consideration of the patient’s characteristics. Additionally, it is important to achieve circumferential fusion including both posterolumbar interbody fusion and posterolateral fusion in a lumbar spinal fusion. This study is focused on the fusion rates of spinal stenosis or degenerative spondylolisthesis less than grade using two kinds of DBM/local autologous bone composite. Therefore, when multilevel fusion surgery is planned, we recommend circumferential fusion be performed by both posterolateral fusion and interbody fusion, regardless of fusion materials.

In this study, the fusion rate was slightly lower than that noted in previous reports [8, 11, 14], which likely reflected our strict criteria for bone union. Measurements were obtained from dynamic radiographs for a definitive evaluation of the fusion status. We defined pseudarthrosis as angular motion < 3° and 2 mm of sagittal motion at a Cobb angle on postoperative flexion/extension radiographs. Because the instrumentation used in this study might have inhibited motion on the dynamic radiographs, we also assessed the CT images for a fusion mass. CT scanning has superior sensitivity to bone growth in the graft area compared with simple radiographs and offers useful visualization of growing bone bridges [43]. In this study, we collected coronal and sagittal images of patients to identify whether the cranial and caudal endplates were connected by vertical bone bridges [10]. When interpreting the fusion status using CT scans, most previous studies have focused on the presence and continuity of bone bridges [10–12]. In patients who had more than one-level fusions, each level was evaluated independently for segment fusion, and the entire set of levels had to show continuity in the fusion mass to be considered overall fusion, while the observation of a one-level pseudarthrosis was considered unfused [10–12].

This study had some limitations. The sample size was relatively small to provide a meaningful analysis and the comparative analysis overestimates the differences between the two groups. However, to the best of our knowledge, this is the first study to perform side-by-side comparisons of fusion efficacy between two DBMs/local autograft composites for graft extension and enhancement after instrumented PLF with unusual peculiar clinical model. Moreover, the self-control design of this study, wherein two different DBMs were implanted on each side in the same patient, eliminated the effects of patient comorbidities, such as DM, smoking, and obesity, which is a great advantage.

## Conclusion

The fusion rates of two kinds of DBMs/local autograft composites was similar of about 70% with improvement of clinical symptoms for lumbar spinal stenosis or degenerative spondylolisthesis less than grade 1. Our results suggest that two kinds of DBMs/local autograft composites might be considered as useful bone graft substitute in instrumented posterolateral fusion for lumbar spinal stenosis or degenerative spondylolisthesis less than grade 1.

## Data Availability

The datasets used and/or analyzed during the current study are available from the corresponding author on reasonable request.

## Abbreviations

ICBG: Iliac crest bone graft
DBM: Demineralized bone matrix
BMP: Bone morphogenetic proteins
PLF: Posterolateral lumbar fusion
CT: Computed tomography
MRI: Magnetic resonance imaging
DM: Diabetes mellitus
BMI: Body mass index
VAS: Visual analog scale
ODI: Oswestry Disability Index
